# Impact of implementation of 2019 European respiratory distress syndrome guidelines on bronchopulmonary dysplasia in very preterm infants

**DOI:** 10.1186/s13052-024-01752-4

**Published:** 2024-09-16

**Authors:** Chongbing Yan, Xiaohui Gong, Hao Luo, Yibo Liu, Yating Lin, Bowen Weng, Cheng Cai

**Affiliations:** Department of Neonatology, Shanghai Children’s Hospital, School of Medicine, Shanghai Jiao Tong University, Shanghai, China

**Keywords:** Bronchopulmonary dysplasia, Respiratory distress syndrome, Very preterm infants, Quality improvement, Outcome

## Abstract

**Background:**

To evaluate the impact of implementation of 2019 European respiratory distress syndrome (RDS) guidelines on the incidence of bronchopulmonary dysplasia (BPD).

**Method:**

We retrospectively collected the clinical data of very preterm infants (VPIs) born before 32 gestational weeks from January 1st 2018 to December 31st 2021. VPIs were divided into group A and group B according to their birth date which was before or at/after January 1st 2020, when the 2019 European RDS guidelines were introduced. BPD is considered as primary outcome. We statistically analyzed all the data, and we compared the general characteristics, ventilation support, medication, nutrition and the outcomes between the two groups.

**Results:**

A total of 593 VPIs were enrolled, including 380 cases in group A and 213 cases in group B. There were no statistic differences regarding to gender ratio, gestational age, birth weight and delivery mode between the two groups. Compared with group A, group B showed higher rate of antenatal corticosteroid therapy (75.1% vs. 65.5%). The improvement of ventilation management in these latter patients included lower rate of invasive ventilation (40.4% vs. 50.0%), higher rate of volume guarantee (69.8% vs. 15.3%), higher positive end expiratory pressure (PEEP) [6 (5, 6) vs. 5 (5, 5) cmH_2_O] and higher rate of synchronized nasal intermittent positive pressure ventilation (sNIPPV) (36.2% vs. 5.6%). Compared with group A, group B received higher initial dose of pulmonary surfactant [200 (160, 200) vs. 170 (130, 200) mg/Kg], shorter antibiotic exposure time [13 (7, 23) vs. 17 (9, 33) days], more breast milk (86.4% vs. 70.3%) and earlier medication for hemodynamically significant patent ductus arteriosus (hsPDA) treatment [3 (3, 4) vs. 8 (4, 11) days] (*p* < 0.05). As the primary outcome, the incidence of BPD was significantly decreased (16.9% vs. 24.2%) (*p* < 0.05), along with lower extrauterine growth retardation (EUGR) rate (39.0% vs. 59.7%), while there were no statistic differences regarding to other secondary outcomes, including mortality, intraventricular hemorrhage (IVH), periventricular leukomalacia (PVL), retinopathy of preterm (ROP) and necrotizing enterocolitis (NEC). However, in the subgroups of infants less than 28 gestational weeks or infants less than 1,000 g, the incidence of BPD was not significantly decreased (*p* > 0.05).

**Conclusions:**

After implementation of 2019 European RDS guidelines, the overall incidence of BPD was significantly decreased in VPIs. Continuous quality improvement is still needed in order to decrease the incidence of BPD in smaller infants who are less than 28 gestational weeks or less than 1,000 g.

**Supplementary Information:**

The online version contains supplementary material available at 10.1186/s13052-024-01752-4.

## Background

Following the improvement of antenatal-perinatal medicine, maternal and child healthcare, neonatal resuscitation skills and integrated preterm management, the survival rate of preterm infants has significantly increased in the last 30 years worldwide [1–3]. Unfortunately, some of the very preterm infants (VPIs) who are delivered before 32 gestational weeks may develop different kinds of on-going problems, including bronchopulmonary dysplasia (BPD), the most common and severe respiratory complication of VPIs [4], which is notably associated with poor neurodevelopmental outcomes [5]. According to data from Chinese Neonatal Network (CHNN), the overall incidence of BPD in Chinese VPIs is about 29.2% [6]. However, in extremely preterm infants (EPIs) less than 28 gestational weeks the incidence of BPD could be significantly higher [7].

European consensus guidelines on management of respiratory distress syndrome (RDS) were published in 2007 for the first time and have been updated every 3 years for several times [8]. They were translated into Chinese and published in national medical journal, to provide theoretical and practical guidance for Chinese neonatologists to manage preterm infants at early stage postnatally. However, due to uneven economic conditions and medical resources, there is wide variation of preterm care practices and subsequent neonatal outcomes in different regions in China [9].

As one of the participants of CHNN [10], we have been improving our institutional management of VPIs within the quality improvement structure of the national network based on our own fundamental conditions. In accordance with 2019 updated version of European RDS guidelines [11] and domestic literature recommendations, we modified and initiated our protocol of VPIs management since January 2020, including ventilation support, medication, nutrition, treatment of patent ductus arteriosus (PDA) and so on. This study is aiming to analyze the impact of the modified practices on the incidence of BPD and other neonatal outcomes.

## Patients and methods

### Study design and objects

Inclusion criteria: All of the infants born less than 32 gestational weeks admitted to the Department of Neonatology in Shanghai Children’s Hospital from January 1st 2018 to December 31st 2021 were screened for this retrospective single-center cohort study.

The exclusion criteria were: (1) early death within 14 days after birth, because these patients could not be diagnosed with BPD based on the definition, (2) withdrawing medical support or discharge against medical advice (DAMA) within 14 days after birth, (3) admission after 14 days after birth, (4) complex congenital heart disease, chromosomal abnormality or gene mutation which may impact long-term outcomes, (5) repeated admission after discharge.

**Fig. 1:**
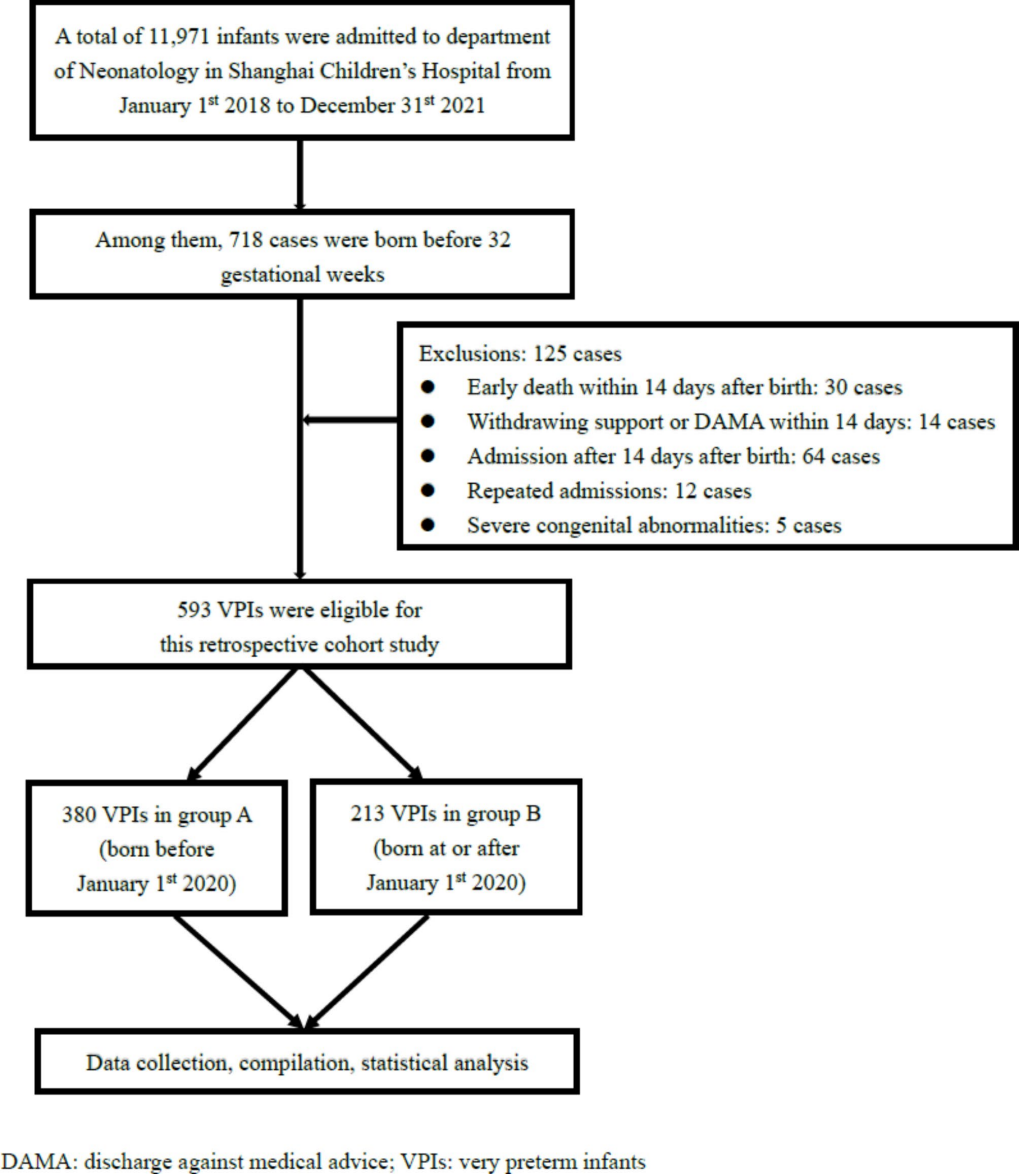
Patients enrollment flowchart

Enrolled infants were divided into group A and group B according to their birth date, which was before or at/after January 1st 2020 when the 2019 European RDS guidelines were introduced, respectively. Data including maternal history, gestational age (GA), birth weight (BW), postnatal management, main inspection results and outcomes were collected (Fig. 1).

The institutional management protocol of VPIs was modified and initiated since January 1st 2020. The main changes were based on European consensus guidelines on management of RDS which were published in 2019, including improvement of ventilation management, administration of pulmonary surfactant (PS), nutrition, medication for infection, sedation and PDA management. All the data were statistically analyzed, and the general characteristics, ventilation support, medication, nutrition and the outcomes between the two groups were compared.

### Ethics

This study was reviewed and approved by ethic committee of Shanghai Children’s Hospital in accordance with the Declaration of Helsinki (approval number: 2021R013-E01). All the parents of enrolled patients read and signed the informed consent at admission that their children’s clinical data other than their personal information might be used for clinical statistical analysis for publication.

### Primary outcome

BPD was considered as the primary outcome for this cohort. Due to lack of universal diagnostic criteria, different studies may use different definitions and classifications to diagnose BPD. It is reported that different standards of diagnosis for BPD could impact the statistical result [12–14]. Based on our clinical practices and understanding to BPD, we adopted the 2018 revised BPD definition proposed by the National Institute of Child Health and Human Development (NICHD) in this retrospective cohort study [15] (supplementary material 1).

### Secondary outcomes

The secondary outcomes included death, intraventricular hemorrhage (IVH) (grade ≥ 3), periventricular leukomalacia (PVL), retinopathy of prematurity (ROP) (stage ≥ 3 or any need for intervention), necrotizing enterocolitis (NEC) (stage ≥ 2) and extrauterine growth restriction (EUGR).

IVH was defined as equal to or greater than grade 3 according to Papile criteria [16]. PVL was defined as presence of periventricular cysts either identified by cranial ultrasound or magnetic resonance imaging (MRI). ROP was defined as equal to or greater than stage 3 or any need for intervention according to International Classification of Retinopathy of Prematurity [17]. NEC was defined as equal to or greater than stage 2 according to modified Bell’s staging criteria [18]. EUGR was defined as the Z score of body weight at the time of discharge decreased > 1.0 compared to which at the time of birth.

### Other definitions

Admission hypothermia was defined as axillary temperature < 36.0 Celsius degree at admission. Prolonged premature rupture of membrane (PPROM) was defined as membrane rupture ≥ 18 h before delivery. Intensive resuscitation was defined as any demand of positive pressure ventilation, intubation, chest compression or epinephrine during birth resuscitation. High fraction of inspired oxygen (FiO_2_) demand was defined as FiO_2_ demand > 0.6 more than 6 h continuously at any time. Early onset sepsis was defined as clinically diagnosed or pathogen proven sepsis within 3 days after birth. Late onset sepsis was defined as clinically diagnosed or pathogen proven sepsis which occurred after 3 days after birth. Long-term sedation was defined as continuous sedation demand of fentanyl or midazolam for over 3 consecutive days. The definition of hemodynamically significant PDA (hsPDA) referred to literature published by Shepherd [19] (supplementary material 2).

### Statistical analysis

Categorical variables were presented by percentages. All continuous variables received test for distribution. Continuous variables with normal distribution were expressed as mean ± standard deviation (SD). Continuous variables with non-normal distribution were described as median (Q1, Q3). χ^2^ test was performed for numeration data and independent-samples *t* test or Mann-Whitney U test was performed for measurement data. All the statistical analyses were performed using SPSS Statistics 27.0 (IBM, US). *p* < 0.05 was considered to indicate a statistically significant difference.

## Results

### General characteristics

A total of 593 VPIs were enrolled in this retrospective cohort study, including 380 cases in group A and 213 cases in group B. There was no statistic difference regarding to gender ratio, gestational age, birth weight, conception and delivery mode, and Apgar score at the 5th minute between the two groups (*p* > 0.05), while group B showed significantly higher rate of gestational diabetes mellitus (GDM) (17.3% vs. 11.6%), antenatal corticosteroid administration (75.1% vs. 65.5%) and magnesium sulfate therapy (68.1% vs. 50.8%), with lower rate of admission hypothermia (16.0% vs. 35.5%) (*p* < 0.05) (Table 1).

**Table 1:** Comparison of general characteristics between group A and group B

Variables *n* (%)	Group A (*n* = 380)	Group B (*n* = 213)	Z / χ^2^	*p* value
Gender Male	227 (59.7)	111 (52.1)	3.237	0.072
Gestational Age (weeks)	30.0 (29.0, 31.3)	30.4 (29.1, 31.1)	-0.111	0.912
Birth Weight (g)	1380 (1185, 1600)	1410 (1135, 1625)	-0.090	0.928
IVF	118 (31.1)	54 (25.4)	2.154	0.142
**Gestational complications**				
Gestational hypertension	52 (13.7)	36 (16.9)	1.118	0.290
GDM	44 (11.6)	37 (17.3)	3.883	0.049*
PPROM	104 (27.4)	43 (20.2)	3.775	0.052
FGR	11 (2.9)	10 (4.7)	1.295	0.255
**Antenatal interventions**				
Antibiotics	134 (35.3)	71 (33.3)	0.225	0.635
Corticosteroid	249 (65.5)	160 (75.1)	5.867	0.015*
Magnesium sulfate	193 (50.8)	145 (68.1)	16.639	< 0.001*
**Delivery history**				
Cesarean section	251 (66.1)	129 (60.1)	1.787	0.181
Intensive resuscitation	116 (30.5)	75 (35.2)	1.372	0.241
Apgar score (5th minute)	9 (8, 9)	9 (8, 9)	-0.030	0.976
Admission hypothermia	135 (35.5)	34 (16.0)	25.637	< 0.001*

IVF, in-vitro fertilization; GDM, gestational diabetes mellitus; PPROM, prolonged premature rupture of membrane; FGR, fetal growth restriction

* *p* < 0.05 indicates a statistically significant difference

### Risk factors of BPD

The overall incidence of BPD for the whole cohort was 21.6% (128/593). The risk factors of BPD included birth weight less than 1,000 g, gestational age less than 28 weeks, male gender, fetal growth restriction (FGR), intensive resuscitation, invasive mechanical ventilation (IMV), high FiO_2_ demand, hypercarbia, sepsis, surfactant demand, long-term sedation, feeding intolerance, hsPDA and EUGR, while Cesarean section seemed to be a protective factor for BPD (Table 2).

**Table 2:** Overall risk factors of BPD

Risk factors *n* (%)	Non-BPD (*n* = 465)	BPD (*n* = 128)	OR	95% CI
**General risks**				
BW < 1,000 g	20 (4.3)	35 (27.3)	8.374	4.627, 15.153*
GA < 28 weeks	30 (6.5)	35 (27.3)	5.457	3.191, 9.332*
Male infants	255 (54.8)	83 (64.8)	1.519	1.012, 2.280*
IVF	137 (29.5)	35 (27.3)	0.901	0.582, 1.395
**Antenatal risks**				
Gestational hypertension	64 (13.8)	24 (18.8)	1.446	0.863, 2.423
GDM	63 (13.5)	18 (14.1)	1.044	0.594, 1.837
PPROM	110 (23.7)	37 (28.9)	1.312	0.847, 2.033
FGR	12 (2.6)	9 (7.0)	2.855	1.175, 6.935*
Antenatal corticosteroid	323 (69.5)	86 (67.2)	0.900	0.592, 1.368
Antenatal magnesium sulfate	267 (57.4)	71 (55.5)	0.924	0.623, 1.370
**Birth risks**				
Cesarean section	309 (66.5)	71 (55.5)	0.629	0.422, 0.936^#^
Intensive resuscitation	111 (23.9)	80 (62.5)	5.315	3.505, 8.062*
Admission hypothermia	130 (28.0)	39 (30.5)	1.129	0.736, 1.731
**Postnatal risks**				
IMV	170 (36.6)	106 (82.8)	8.361	5.089, 13.736*
High FiO_2_ demand	12 (2.6%)	37 (28.9)	15.349	7.707, 30.569*
Hypercarbia	9 (1.9)	22 (17.2)	10.516	4.707, 23.492*
EOS	54 (11.6)	25 (19.5)	1.847	1.097, 3.110*
LOS	80 (17.2)	46 (35.9)	2.700	1.749, 4.167*
Surfactant demand	282 (60.6)	109 (85.2)	3.723	2.210, 6.272*
Long-term sedation	38 (8.2)	53 (41.4)	7.941	4.896, 12.878*
Feeding intolerance	256 (55.1)	111 (86.8)	5.331	3.100, 9.167*
hsPDA	53 (11.4)	62 (48.4)	7.302	4.659, 11.446*
EUGR	224 (48.2)	86 (67.2)	2.203	1.460, 3.324*

BPD, bronchopulmonary dysplasia; BW, birth weight; GA, gestational age; IVF, in-vitro fertilization; GDM, gestational diabetes mellitus; PPROM, prolonged premature rupture of membrane; FGR, fetal growth restriction; IMV, invasive mechanical ventilation; EOS, early onset sepsis; LOS, late onset sepsis; hsPDA, hemodynamically significant patent ductus arteriosus; EUGR, extrauterine growth restriction

^#^ indicates protective factors of BPD; * indicates high risk factors of BPD

### Postnatal management

Based on 2019 European consensus of BPD management guidelines, several changes were achieved in clinical practices for VPIs in group B compared with group A (Table 3). For respiratory support, IMV (40.4% vs. 50.0%), high flow nasal cannula (HFNC) (26.5% vs. 43.4%) and nasal high frequency oscillatory ventilation (nHFOV) (3.7% vs. 9.4%) were used less, volume guarantee (VG) (69.8% vs. 15.3%) and synchronized nasal intermittent positive pressure ventilation (sNIPPV) (36.0% vs. 5.3%) were used more with higher initial positive end expiratory pressure (PEEP) [6 (5, 6) vs. 5 (5, 6) cmH_2_O] or continuous positive airway pressure (CPAP) [6 (5, 6) vs. 5 (5, 5) cmH_2_O]. The overall rate of PS administration was similar, but the initial dose was higher in group B [200 (160, 200) vs. 170 (130, 200) mg/Kg], with a lower rate of long-term sedation (9.9% vs. 18.4%) and shorter antibiotics exposure time [13 (7, 23) vs. 17 (9, 33) days]. For nutrition management, group B showed higher rate of early initiation of enteral feeding, breast milk, donor milk and fortifier usage. For PDA management, the overall rate of medication or ligation for hsPDA was the same, but group B was treated earlier with more ibuprofen.

### Outcomes

The incidence of BPD was significantly lower in group B, compared with group A (16.9% vs. 24.2%) (*p* < 0.05). However, there was no statistical difference regarding to severity of BPD between the two groups.

As for secondary outcomes, less infants in group B developed EUGR, while there was no statistical difference in mortality, incidence of IVH, PVL, ROP and NEC between the two groups (Table 4).

### Subgroups comparison

After dividing patients into subgroups based on gestational weeks or birth weight, there was no significant difference of BPD in extremely preterm infants (EPIs) (GA < 28 weeks) or extremely low birth weight infants (ELBW) (BW < 1,000 g) (Table 5).

**Table 3:** Comparison of management between group A and group B

Management *n* (%)	Group A (*n* = 380)	Group B (*n* = 213)	Z / χ^2^	*p* value
**Respiratory support**				
IMV	190 (50.0)	86 (40.4)	5.083	0.024*
Volume guarantee	29 (15.3)	60 (69.8)	80.499	< 0.001*
HFOV	26 (13.7)	8 (9.3)	1.052	0.305
Initial PEEP (cmH_2_O)	5 (5, 6)	6 (5, 6)	-3.968	< 0.001*
Non-invasive ventilation	339 (89.2)	189 (88.7)	0.032	0.858
CPAP	314 (92.6)	180 (95.2)	1.375	0.241
sNIPPV	18 (5.3)	68 (36.0)	83.710	< 0.001*
HFNC	147 (43.4)	50 (26.5)	14.831	< 0.001*
nHFOV	32 (9.4)	7 (3.7)	5.836	0.016*
Initial CPAP (cmH_2_O)	5 (5, 5)	6 (5, 6)	-11.486	< 0.001*
**Medication**				
PS	255 (67.1)	136 (63.8)	0.644	0.422
PS first dose (mg/kg)	170 (130, 200)	200 (160, 200)	-4.101	< 0.001*
Caffeine	356 (93.7)	206 (96.7)	2.528	0.112
Antibiotics exposure (d)	17 (9, 33)	13 (7, 23)	-3.583	< 0.001*
Intravenous steroid	18 (4.7)	9 (4.2)	0.082	0.774
Inhaled steroid	133 (35.0)	65 (30.5)	1.234	0.267
Diuretics	94 (24.7)	48 (22.5)	0.363	0.547
Long-term sedation	70 (18.4)	21 (9.9)	7.702	0.006*
**Nutrition**				
Enteral feeding within 24 h	272 (71.6)	178 (83.6)	10.721	0.001*
Breast milk	267 (70.3)	184 (86.4)	19.480	< 0.001*
Donor milk	321 (84.5)	197 (92.5)	7.936	0.005*
Fortifier	277 (72.9)	184 (86.4)	14.354	< 0.001*
**PDA management**				
Any PDA	264 (69.5)	147 (69.0)	0.014	0.907
hsPDA	73 (27.7)	42 (28.6)	0.119	0.730
Medication	63 (23.9)	42 (28.6)	1.100	0.294
Ibuprofen/Paracetamol	46/20	42/4	7.517	0.006*
Initiation of medication (d)	8 (4, 11)	3 (3, 4)	-5.998	< 0.001*
Ligation	6 (2.3)	2 (1.4)	0.412	0.521

IMV, invasive mechanical ventilation; HFOV, high frequency oscillatory ventilation; PEEP, positive end expiratory pressure; CPAP, continuous positive airway pressure; sNIPPV, synchronized nasal intermittent positive pressure ventilation; HFNC, high flow nasal cannula; nHFOV, nasal high frequency oscillatory ventilation; PS, pulmonary surfactant; PDA, patent ductus arteriosus; hsPDA, hemodynamically significant patent ductus arteriosus

* *p* < 0.05 indicates a statistically significant difference

**Table 4:** Comparison of outcomes between group A and group B

Outcomes *n* (%)	Group A(*n* = 380)	Group B (*n* = 213)	χ^2^	*p* value
**Primary outcome**				
BPD	92 (24.2)	36 (16.9)	4.308	0.038*
Grade I	42 (45.7)	15 (41.7)	0.166	0.683
Grade II	33 (35.9)	14 (38.9)	0.102	0.750
Grade III or III(A)	17 (18.5)	7 (19.4)	0.016	0.900
**Secondary outcomes**				
Death	10 (2.6)	2 (0.9)	1.972	0.160
IVH	20 (5.3)	10 (4.7)	0.092	0.762
PVL	17 (4.5)	6 (2.8)	1.005	0.316
ROP	15 (3.9)	3 (1.4)	2.989	0.084
NEC	12 (3.2)	8 (3.8)	0.150	0.699
EUGR	227 (59.7)	83 (39.0)	23.601	< 0.001*

BPD, bronchopulmonary dysplasia; IVH, intraventricular hemorrhage; PVL, periventricular leukomalacia; ROP, retinopathy of prematurity; NEC, necrotizing enterocolitis; EUGR, extrauterine growth restriction

* *p* < 0.05 indicates statistically significant difference

**Table 5:** Comparison of incidence of BPD in subgroups

Subgroups *n* (%)	Group A		Group B		χ^2^	*p* value
	BPD	Non-BPD	BPD	Non-BPD		
GA < 28 weeks	24 (55.8)	19 (44.2)	11 (50.0)	11 (50.0)	0.198	0.656
GA ≥ 28 weeks	68 (20.2)	269 (79.8)	25 (13.1)	166 (86.9)	4.222	0.040*
BW < 1,000 g	23 (71.9)	9 (28.1)	12 (52.2)	11 (47.8)	2.245	0.134
BW ≥ 1,000 g	69 (19.8)	279 (80.2)	24 (12.6)	166 (87.4)	4.451	0.035*

BPD, bronchopulmonary dysplasia; GA, gestational age; BW, birth weight

* *p* < 0.05 indicates statistically significant difference

## Discussion

BPD is a major complication which impacts the long-term outcomes of VPIs. One of the most important tasks for neonatologists to manage VPIs is aiming to reduce the incidence of BPD and subsequent neurodevelopment disorders. For this purpose, we adopted 2019 European consensus of RDS management guidelines and improved our clinical practices since January 2020, including optimized respiratory support and medication use, improved nutrition management and timely intervention for hsPDA. As a result, the overall incidence of BPD in VPIs was decreased significantly (16.9% vs. 24.2%).

It is well known that smaller gestation age and lower birth weight are high risk factors of BPD. In a previous study, it was reported that oxygen during resuscitation, surfactant demand, NIPPV, IMV, HFOV, PDA and nosocomial sepsis were also associated with BPD in preterm infants born at or less than 32 weeks of gestation [20]. In addition, higher FiO2 demand and maximum mean airway pressure (MAP) were reported as risks of BPD in EPIs [21]. In our cohort, we also found that BPD is significantly associated with FGR, long-term sedation, feeding intolerance and EUGR. FGR is considered as a pattern of fetal programming which could impact the long-term outcomes [22]. It is reported that FGR could increase the incidence of BPD with hypothesis of mechanistic link between fetal programming and vascular architecture and mechanics [23]. Antenatal corticosteroid exposure is considered to decrease mortality rate in EPIs, but the rate of BPD in survivors did not differ [24]. In our cohort, we also found that BPD was not associated with antenatal corticosteroid exposure. However, it is obvious that the rate of antenatal corticosteroid treatment in our preterm population had been increasing over recent years (75.1% vs. 65.5%), although it is still lower than the number reported in high-income countries which is usually over 90%.

In order to decrease the incidence of BPD, we modified our protocol of management of VIPs in accordance with 2019 European RDS guidelines. Protective ventilation strategy is essential to protect fragile lungs of VPIs from pulmonary volutrauma and barotrauma. It has been proven that prophylactic nasal CPAP in very preterm infants can reduce the incidence of BPD compared to mechanical ventilation [25]. NIPPV could reduce incidence of extubation failure and need for reintubation, although it has no effect on BPD or mortality [26]. In addition, synchronization is very important to use NIPPV. If neonates need mechanical ventilation, volume targeted ventilation mode could reduce the rate of death or BPD, pneumothoraces, hypocarbia, severe neurological outcomes and duration of ventilation, compared with pressure limited ventilation mode [27]. Elective HFOV could slightly reduce the risk of BPD compared with conventional ventilation, but the evidence is weak due to inconsistency in the different studies [28]. Therefore, based on above evidence and combined with 2019 European RDS guidelines, we optimized our ventilation strategy in VPIs. The overall rate of IMV was decreased (40.4% vs. 50%). Most infants who needed mechanical ventilation were put on VG mode (69.8% vs. 15.3%) with a higher initial PEEP [6 (5, 6) vs. 5 (5, 6) cm H_2_O]. For non-invasive ventilation mode, sNIPPV was used more (36% vs. 5.3%), while HFNC (26.5% vs. 43.4%) and nHFOV (3.7% vs. 9.4%) were used less. New evidence for better ventilation strategies continuously emerges overtime. Zhu et al. [29] reported that both nHFOV and NIPPV could lower the risk of reintubation compared with CPAP, while they did not find any difference in BPD within those 3 groups. The latest Cochrane Database systemic review pointed out that early NIPPV may reduce the risk of respiratory failure, the need for intubation and the rate of BPD in preterm infants with a gestational age of 28 to 32 weeks [30]. It reminds us that we should continuously optimize our ventilation strategies based on current evidence in order to improve the pulmonary outcomes of VPIs.

Rational medication for VPIs is also important. It has been around 30 years since exogenous PS was introduced to Chinese neonatologists, which saved many lives of preterm infants who might die of RDS. Based on the Cochrane review published in 2015 [31], 2019 European RDS guidelines recommended that porcine sourced PS with an initial dose of 200 mg/kg is better for RDS rescue therapy. Hence, we increased the initial dosage of PS administration in VPIs [200 (160, 200) vs. 170 (130, 200) mg/Kg]. A recent retrospective study found that switching initial dose of PS from 100 mg/kg to 200 mg/kg was associated with a marked reduction of BPD [32]. Empiric antibiotic therapy is considered as a risk factor of BPD in VLBW infants [33]. Therefore, we improved our antibiotics stewardship and significantly decreased the antibiotic exposure time of VPIs [13 (7, 23) vs. 17 (9, 33) days]. Routine use of sedation in VPIs on mechanical ventilation is not recommended [11]. Thereupon, we decreased the use of long-term sedation of fentanyl or midazolam in VPIs (9.9% vs. 18.4%). In respect of other medications, there was no significant difference in the use of caffeine, postnatal steroid and diuretics between the two groups.

Nutrition is another crucial part of management of VPIs. Breast milk is always considered as the best food source for neonates. A systematic review indicated that use of exclusive mother’s own milk feedings was associated with a significant reduction in the risk of BPD in very preterm infants [34]. Another systematic review suggested that donor milk was also effective to protect against BPD [35]. In 2016, our hospital established the first charitable donor milk bank in Shanghai. Due to the benefit from the support of donor milk bank, we are able to provide appropriate enteral nutrition to VPIs in the early stage of life. The improvement of nutrition management of VPIs in our clinical practice included higher rate of breast milk feeding (86.4% vs. 71.6%), donor milk feeding (92.5% vs. 84.5%), human milk fortifier (86.4% vs. 72.9%) and early enteral feeding within 24 h postnatally (83.6% vs. 71.6%). Above measures resulted in a significant reduction of EUGR (39.0% vs. 59.7%).

Whether, when or how to treat a PDA is still a tricky question to neonatologists. In general, hsPDA is considered to be a risk factor in the development of BPD [36]. Previous studies indicate that delaying pharmacologic PDA treatment for 2 ~ 3 days after birth does not increase the incidence of BPD, but delaying treatment after 1 week may be associated with an increase of BPD [37]. With regard to medication for PDA, a systematic review in 2018 pointed out that oral ibuprofen had same rate of PDA closure compared with intravenous indomethacin, along with a significant reduction of risk of NEC [38]. Paracetamol was reported as effective as ibuprofen for PDA closure but the evidence was not strong [39]. Therefore, we aimed to identify hsPDA and start intervention earlier in VPIs. The initiation time of medication for hsPDA was significant earlier in group B, compared with group A [3 (3, 4) vs. 8 (4, 11) days]. We also used more ibuprofen rather than paracetamol. Only 2 infants who failed with pharmacological PDA closure and suffered from persistent hemodynamic impact received ligation surgery in group B.

After we improved our clinical practice on ventilation management, medication, nutrition and PDA treatment based on 2019 European RDS management guidelines, the overall incidence of BPD in VPIs significantly decreased (16.9% vs. 24.2%), along with a lower incidence of EUGR (39% vs. 59.7%). There was no statistic significant difference in other outcomes, such as death, IVH, PVL, ROP and NEC. However, the severity of BPD did not differ. Meanwhile, around half of the smaller preterm infants who were less than 1,000 g or less than 28 gestational weeks still developed BPD despite of all the above quality improvement measures. It reminds us that continuous quality improvement is still needed to reduce the incidence of BPD in smaller preterm infants.

There are some limitations in this study. Firstly, as a children’s hospital without maternal settings, all the patients in our department are out-born. Improvement of antenatal care or resuscitation in the delivery room is outside of our jurisdiction. Secondly, BPD is a multifactorial disease, but some external environmental impacts are not discussed in this study, such as the influence of COVID which seriously impacted the overall birth rate and human resource in medical facilities. Lastly, as a single-center retrospective study, the number of extremely small infants was small, along with a portion of early death or withdrawing treatment cases, which may cause bias of the statistical analysis.

## Conclusions

After implementation of 2019 European RDS guidelines, the overall incidence of BPD was significantly decreased in VPIs. Continuous quality improvement is still needed in order to decrease the incidence of BPD in smaller infants < 28 weeks or < 1,000 g.

## Electronic supplementary material

Below is the link to the electronic supplementary material.

Supplementary Material 1

Supplementary Material 2

## Data Availability

The datasets used and/or analyzed during this study are available from the corresponding author on reasonable request.
