# Supplementary material for: Impact of implementation of 2019 European respiratory distress syndrome guidelines on bronchopulmonary dysplasia in very preterm infants
Source: Ital J Pediatr. 2024 Sep 16;50:178. doi: 10.1186/s13052-024-01752-4 (PMC11407007; doi:10.1186/s13052-024-01752-4)
Supplement: Supplementary file 1 — Supplementary Material 1 [file 13052_2024_1752_MOESM1_ESM.docx]

**Definition and classification of BPD based on 2018 NICHD criteria:**

Preterm infants (born less than 32 gestational weeks) show persistent parenchymal lung disease with radiographic changes at 36 weeks PMA require 1 of following respiratory support for ≥ 3 consecutive days to maintain arterial oxygen saturation in the 90% ~95% range.

| **Grades** | Nasal cannula <1L/min | Nasal cannula 1~3L/min | NCPAP, NIPPV, nHFOV or  HFNC ≥ 3L/min | Invasive ventilation |
| --- | --- | --- | --- | --- |
| **I** | 22%~70% | 22%~29% | 21% | / |
| **II** | >70% | ≥30% | 22%~29% | 21% |
| **III** | / | / | ≥30% | >21% |
| **III(A)** | Early death between 14 days of postnatal age and 36 weeks owing to persistent parenchymal lung disease and respiratory failure that cannot be attributable to other neonatal morbidities | | | |

NCPAP, nasal continuous positive airway pressure; NIPPV, nasal intermittent positive pressure ventilation; nHFOV, nasal high frequency oscillatory ventilation; HFNC, high flow nasal cannula
