# Supplementary material for: Impact of implementation of 2019 European respiratory distress syndrome guidelines on bronchopulmonary dysplasia in very preterm infants
Source: Ital J Pediatr. 2024 Sep 16;50:178. doi: 10.1186/s13052-024-01752-4 (PMC11407007; doi:10.1186/s13052-024-01752-4)
Supplement: Supplementary file 2 — Supplementary Material 2 [file 13052_2024_1752_MOESM2_ESM.docx]

**Echocardiographic features for a hemodynamically significant PDA**

| Echocardiographic indicators | Non-hsPDA | hsPDA |
| --- | --- | --- |
| Ductal features |  |  |
| Diameter (mm) | <1.5 (GA≤26 weeks) | ≥1.5 (GA≤26 weeks) |
|  | <2.0 (GA>26 weeks) | ≥2.0 (GA>26 weeks) |
|  | or | and |
| Shunt pattern | Closing, bidirectional | Growing, pulsatile |
| Pulmonary overflow |  |  |
| LA:Ao | <1.4 | ≥1.4 |
| LVO (ml/kg/min) | ≤300 | >300 |
| LPA EDV (cm/s) | ≤20 | >20 |
| Systemic hypotension |  |  |
| Descending aortic flow | Antegrade | Retrograde |
| Organ blood flow | Normal | Absent/reversed diastolic flow |

PDA, patent ductus arteriosus; hsPDA, hemodynamically significant patent ductus arteriosus; GA, gestational age, LA:Ao, left atrium to aortic root ratio; LPA EDV, left pulmonary artery end-diastolic velocity; LVO, left ventricular output
